# Preclinical Combination Targeting VEGF and PI3K in a Rare, Aggressive Mixed Endometrial Carcinoma: An Applied Case Report

**DOI:** 10.1158/2767-9764.CRC-25-0634

**Published:** 2026-04-15

**Authors:** Nikolina Radulovich, Pamela Soberanis Pina, Molly L. Udaskin, Quan Li, Kevin C.J. Nixon, Irene Y. Xie, Ankita Nand, Joshua C. Rosen, Ming Li, Anjelica Hodgson, Nhu-An Pham, Amit M. Oza, Mathieu Lupien, Robert Rottapel, Ming-Sound Tsao, Stephanie Lheureux

**Affiliations:** Ontario Cancer Institute, Princess Margaret Cancer Centre, University Health Network, Toronto, Canada.

## Abstract

We report a rare case of a young patient (VENUS 167) initially diagnosed with grade 1 endometrioid endometrial cancer, which, following endocrine treatment, presented with mixed aggressive carcinoma with three distinct histologic patterns: grade 1 endometrioid, large cell neuroendocrine, and undifferentiated carcinoma. The surgical specimen at the time of disease progression was used to establish OPTO.85, a patient-derived organoid (PDO), followed by a corresponding organoid-derived xenograft (ODX). Multi-omic analyses confirmed that OPTO.85 accurately reflected the patient’s tumor characteristics. Whole-exome sequencing analysis identified oncogenic alterations in *PIK3CA*, *ARID1A*, and *CTNNB1*. Further RNA sequencing and assay for transposase-accessible chromatin using sequencing analyses revealed enrichment in VEGF and Wnt signaling pathways, suggesting therapeutic vulnerabilities. A high-throughput drug screen was conducted using ApexBio-approved and epigenetic drug libraries, along with kinase inhibitor and tool compound libraries developed at the Ontario Institute of Cancer Research. The OPTO.85 PDO exhibited sensitivity to PI3K inhibitors and responsiveness to VEGF inhibition. Cediranib demonstrated synergy with BKM120, significantly reducing organoid growth. This combination also showed *in vivo* efficacy in the ODX model, in which dual inhibitors significantly suppressed tumor growth compared with single compounds. This case exemplifies the impact of genomic profiling and patient-derived models in identifying actionable molecular changes in rare cancers with limited therapeutic options and poor prognosis. It highlights that high-throughput sequencing for individual patient tumors and generation of patient-derived models are feasible in endometrial cancer. This preclinical model may assist clinical decision and personalized therapy requiring validation in prospective studies.

**Significance:**

This study characterizes a rare aggressive mixed endometrial carcinoma that developed after hormonal therapy. Patient-derived organoid and xenograft models revealed actionable targets in the VEGF and PI3K pathways. Combined cediranib and BKM120 treatment showed synergistic antitumor effects *in vitro* and *in vivo*. These findings highlight the potential of integrating molecular profiling and drug testing to guide personalized therapies in rare and recurrent endometrial cancers.

## Introduction

Endometrial cancer is the most common cancer of the female reproductive organs, encompassing a heterogeneous group of subtypes with distinct features and outcomes (1, 2). Recent advances in molecular profiling have improved risk stratification in endometrial cancer through the development of surrogate molecular subgroups defined by the Proactive Molecular Risk Classifier for Endometrial Cancer (ProMisE) framework. These include POLE-mutated, mismatch repair (MMR)-deficient, p53-abnormal, and no specific molecular profile tumors. The 2023 update to the FIGO (International Federation of Gynecology and Obstetrics) staging system now incorporates these molecular classifications to better inform prognosis and guide treatment decisions (1, 3, 4). With the emergence of precision medicine, endometrial cancer management has shifted toward a molecularly driven, tailored approach. However, histologic subtyping remains challenging, particularly in cases with biphasic or triphasic morphology, in which tumors contain both low- and high-grade carcinoma components. These complex features can negatively affect prognosis, underscoring the need for comprehensive molecular characterization to refine treatment strategies (3, 5).

We present the case of a young patient initially diagnosed with grade 1 endometrioid endometrial carcinoma, which rapidly progressed and later presented as a mixed carcinoma with triphasic morphology. The tumor was refractory to standard treatment, and next-generation panel sequencing revealed oncogenic alterations in *PIK3CA*, *ARID1A*, and *CTNNB1*.

To better understand the disease trajectory and explore potential therapeutic targets, we generated patient-derived organoid (PDO) and organoid-derived xenograft (ODX) models from the patient’s tumor at the time of progression. Through whole-exome sequencing (WES), RNA sequencing (RNA-seq), assay for transposase-accessible chromatin using sequencing (ATAC-seq), and high-throughput drug screening, we identified actionable targets in the vascular endothelial growth factor (VEGF) and phosphoinositide 3-kinase (PI3K) pathways. Drug screening and testing validated the synergistic effects of cediranib and buparlisib (BKM120) in this aggressive endometrial cancer subtype, providing insights into potential treatment strategies.

## Materials and Methods

### Patient samples and clinical data collection

Initial diagnostic tissue, sequential endometrial biopsies, and surgical specimens were collected through the VENUS program (*Study of Biomarkers in Gynecological Cancers*; NCT03420118), which provides genomic profiling data for patients with histologically confirmed gynecologic cancers. The primary objective of the VENUS program is to identify potential biomarkers, assess genomic and immune differences over time, characterize temporal heterogeneity, and track clonal evolution.

Tumor profiling was performed in a College of American Pathologists/Clinical Laboratory Improvement Amendments–accredited laboratory using the University Health Network (UHN) Hi5 panel, a multigene targeted panel assay covering exonic regions of 555 cancer-related genes (6, 7). Clinical data, including demographics, baseline characteristics, and medical history, were also collected and summarized.

The VENUS study was approved by the UHN Research Ethics Board (REB 17-5411.18).

### PDO establishment and characterization

Endometrial cancer tissue was collected at Toronto General Hospital, UHN, under protocols approved by the institutional REBs (REB 17-5411.18 and REB 17-5518). Written informed consent was obtained from all patients prior to participation. All studies involving human participants were conducted in accordance with the Canada Tri-Council Policy Statement on Ethical Conduct for Research Involving Humans and the principles outlined in the Declaration of Helsinki (www.pre.ethics.gc.ca).

Tumor specimens were collected in serum-free Dulbecco’s Modified Eagle Medium (DMEM) and processed within 24 hours. A portion of the tumor was fixed in formalin for histopathologic and immunohistochemical (IHC) analysis, whereas the remaining tissue was minced into 1- to 3-mm pieces. Two random samples were snap-frozen for DNA and RNA extraction.

For organoid generation, the remaining tissue was washed with phosphate-buffered saline and resuspended in 5 mL Advanced DMEM/F12 (Ad-MEM; Gibco) containing collagenase type II and 10 μmol/L Y-27632 (Sigma). Tissue segments were digested at 37°C with agitation for 20 minutes, manually disrupted, and incubated for another 10 minutes. The suspension was filtered through a 40-μm strainer, washed with 10 mL Ad-MEM, and centrifuged at 300 × *g* for 5 minutes. The cell pellet was plated in 50 μL GFR Matrigel (Corning) domes at a seeding density of 80,000 cells per well in a 24-well NUNC plate. Each well was overlaid with 500 μL O63 organoid media, and plates were incubated at 37°C. O63 media includes Ad-MEM medium supplemented with HEPES (1×, Invitrogen), Glutamax (1×, Invitrogen), antibiotic–antimycotic (100 μ/mL, Invitrogen), B27 (1×, Invitrogen), N-acetyl-L-cysteine (1.25 mmol/L, Sigma), human Noggin (100 ng/mL, Peprotech), human EGF (20 ng/mL, Peprotech), human FGF10 (100 ng/mL, Peprotech), human β-FGF (100 ng/mL, Peprotech), Forskolin (10 mm; Sigma), nicotinamide (1 mmol/L, Sigma), Y-27632 (10 mm, Sigma), and β-estradiol (100 nmol/L, Sigma).

Organoid cultures were maintained with media changes every 3 to 4 days and passaged every 7 to 21 days. For passaging, organoid media were aspirated, and Matrigel domes were disrupted using 1 mL TrypLE (Gibco) per well. Plates were incubated at 37°C for 20 to 30 minutes, followed by manual pipetting to dissociate cells. Single cells and small clusters were washed with DMEM/F12, pelleted at 300 × g for 5 minutes, and replated in fresh 50 μL Matrigel domes. After solidifying at 37°C for 10 minutes, 500 μL of organoid media were added.

High-grade serous ovarian carcinoma (HGSOC) organoids and associated data were obtained from Princess Margaret Living Biobank PDO (PMLBPDO) core (RRID: SCR_027530) at UHN, Canada.

### Histologic analysis and IHC

IHC staining was conducted on Ventana Discovery Ultra with primary antibodies synaptophysin (Abcam; ab32127, 1:1,000, RRID: AB_2286949), CAM5.2 (Ventana; 790-4555, auto-dispensed), NCAM1 (Abcam; ab75813, 1:100, RRID: AB_2632384), estrogen receptor (ER; Abcam; AB16660, 1:100, RRID: AB_443420), progesterone receptor (PR; Dako; M3569, clone PgR363, 1:200), and P53-D07 (Dako; M7001, 1:250). Secondary antibodies used were HRP Multimer (Roche Diagnostics; cat. #253-429) and OmniMap antiRabbit HRP (Roche Diagnostics; cat. #760-4311). Whole slides of hematoxylin and eosin histology sections were digitally scanned with 20× objective (Aperio AT2 brightfield scanner, Leica Biosystems Inc.).

### Whole-genome and -exome sequencing and analyses

WES and whole-genome sequencing (WGS) raw sequencing reads were aligned to the human reference genome (GRCh37) using Burrows-Wheeler Aligner v0.7.12 (RRID: SCR_010910; ref. 8). Quality control, local realignment around insertions/deletions (indel), base quality score recalibration, duplicate marking, and further processing of mapped reads were carried out using the standard Genome Analysis Toolkit (GATK) pipeline (v3.4; RRID: SCR_001876; ref. 9) and Picard v1.140 (RRID: SCR_006525; https://broadinstitute.github.io/picard/). Binary Alignment Map files generated from these pipelines were used for variant calling. Somatic single-nucleotide variants and indels were identified using Mutect (RRID: SCR_000559) v1.1.5 and Varscan (RRID: SCR_006849) v2.3.8 (10). Final mutation calls were annotated with Annovar (RRID: SCR_012821) and Variant Effect Predictor (RRID: SCR_007931) v87 (11). Copy-number variation analysis from whole-genome data was performed using Control-FREEC (RRID: SCR_010822) v11.6 (12), and gene-level copy-number status was determined with GISTIC2.0 (13). Mutation data visualization and oncoprint generation were conducted using the R package “ComplexHeatmap” (14).

### RNA-seq and analysis

Total RNA was quality-checked via BioAnalyzer (Agilent 5400), NanoDrop, and agarose gel electrophoresis. Library preparation was performed using AB Clonal Fast RNA-seq Lib Prep Kit V2 for Illumina.pdf. RNA was sequenced using NovaSeq 6000 sequencer with 150-cycle paired-end protocol and multiplexing to obtain 90 to 170 million reads/sample.

Raw fastq reads were aligned to the human genome (hg38) using STAR (RRID: SCR_004463; v. 2.7.9a; ref. 15). In the case of patient-derived xenograft (PDX) samples, reads were first aligned to the mouse genome (mm10) to identify any mouse RNA molecules, followed by the alignment of unaligned reads to the human genome (hg38). Poorly aligned and multimapped reads were filtered prior to generating gene-level counts using featureCounts (RRID: SCR_012919; subread v. 2.0.1; ref. 16). Read counts were imported into R (v. 4.2.0) and normalized prior to differential expression analysis using DESeq2 (RRID: SCR_000154; v. 1.38.3; ref. 17). Differentially expressed genes were defined as genes with a false discover rate (FDR) <0.05 and a fold change (FC) in expression between HGSOC and OPTO.85 samples >2. Gene ontology (GO) enrichment analysis for biological processes was performed on differentially expressed genes using the gprofiler2 Bioconductor package (RRID: SCR_018190; v. 0.2.3; ref. 18).

### ATAC-seq and analysis

Samples were prepared as described previously (19). Briefly, 60,000 viable cells per sample were pelleted at 500 RCF, 4°C for 5 minutes and then resuspended in 50 μL of cold resuspension buffer (RSB) containing 0.1% NP-40, 0.1% Tween-20, and 0.01% digitonin. After a 3-minute incubation on ice, cells were washed with 1 mL cold RSB (0.1% Tween-20), pelleted again, and resuspended in 50 μL of transposition mix. Transposition was carried out at 37°C for 30 minutes at 1,000 RPM. Reactions were purified (Qiagen MinElute) and eluted in 20 μL water. Quantitative PCR (qPCR) determined the optimal PCR cycle number, after which the remaining sample was amplified. Libraries were cleaned twice with AMPure XP beads using a 0.7x–1× double-sided protocol to remove large fragments.

Purified libraries were evaluated for enrichment by qPCR using primers designed against open regions (KAT6B and GAPDH) compared against closed regions (QML93 and SLC22A3). Samples that had a fold enrichment greater than 10 were sequenced. The libraries were quantified by qPCR and then normalized and pooled to 1.25 nmol/L. Each 1.25 nmol/L pool was denatured using 4 μL of 0.2N NaOH (Sigma) for 8 minutes at room temperature (RT) before being neutralized with 5 μL of 400 mmol/L Tris-HCl (Sigma). The neutralized pool was loaded immediately onto a NovaSeq 6000 SP flow cell. Samples were sequenced with the following run parameters: read 1 to 50 cycles, read 2 to 50 cycles, index 1 to 8 cycles, and index 2 to 0 cycles to achieve ∼60 million reads per sample.

To identify OPTO.85-specific and shared peaks, narrowPeak files were compared using bedtools intersect and bedtools merge (RRID: SCR_006646; v. 2.26.0; refs. 20, 21). Peaks were annotated to genomic features using the ChIPseeker Bioconductor package (RRID: SCR_021322; v. 1.34.1; ref. 22). To assess enrichment of pathways in regions with measured accessibility, the genomic regions enrichment of annotations tool (GREAT) analysis was performed using a custom R script to identify significantly enriched GO gene sets from g:profiler (RRID: SCR_006809; https://biit.cs.ut.ee/gprofiler/gost). Deeptools (RRID: SCR_016366; v. 3.5.1; ref. 13) was used to heatmaps and metagene profiles displaying normalized ATAC-seq signal.

### High-throughput drug screening and candidate selection

Organoids were passaged to single cells as described above and robotically dispensed to a 1,536-well plate (Corning 3893BC) at a seeding density of 500 cells/well in 2 μL of 50% Matrigel/media slurry overlaid with 8 μL of organoid media on day 0. We utilized several drug libraries, including ApexBio FDA-Approved Drug Library, (Selleckchem cat. #L130), ApexBio Epigenetic Drug Library, (Selleckchem cat. #L1900), and OICR Kinase Inhibitor and Tool Compound Libraries. Drugs were added on day 3 via an acoustic dispenser (Beckham Echo) in singlicate at 2.5 μmol/L final concentration. Cell viability was assessed by Alamar Blue HS Cell Viability Reagent (Thermo Fisher Scientific, A50101) on day 7. Viability values were normalized to untreated wells. Z′ (Z-prime) for all plates was >0.5. Responses were aggregated by known drug targets and compared with drug responses for cell lines of uterine origin screened in the PRISM study (14).

### *In vitro* drug sensitivity curves

Organoids are processed to single cell as described above. Cells are plated in a 384-well plate (Griener; 781098) at a seeding density of 2,000 cells/well on precoated Matrigel wells; three sets of wells in triplicate were also plated to determine doubling time. All pharmaceutical agents were added the next day, in a 21-point concentration series from 10 to 0.001 μmol/L to wells in triplicate. Cell viability was assessed by ATP quantification using the CellTiter-Glo 3D luminescence-based assay (cat. #G9681, Promega Corp.). Viability values were normalized to vehicle control wells, and dose concentrations were log_10_-transformed. Drug dose–response scores were calculated using area under the curve (AUC) values for each drug and were normalized by dividing the AUC value by the maximum area for the concentration range measured for each drug.

### Immunoblotting analysis

Organoids were lysed in RIPA lysis buffer (Sigma-Aldrich) supplemented with cOmplete, Mini, EDTA-free Protease Inhibitor Cocktail Tablets (Roche) and Pierce Phosphatase Inhibitor Mini Tablets (Thermo Fisher Scientific). Protein concentration was determined using a Pierce BCA Protein Assay Kit (Thermo Fisher Scientific). RIPA lysis buffer and 4× Laemmli sample buffer (Bio-Rad) were added to samples and were boiled at 95°C for 5 minutes for denaturing. Samples were electrophoresed on a 4% to 20% mini-PROTEAN TGX gel (Bio-Rad) and transferred onto nitrocellulose membranes using a Trans-Blot Turbo transfer machine (Bio-Rad). Membranes were blocked in 5% bovine serum albumin (Bio-Rad) in 1× Tris-buffered saline and polysorbate 20 (TBST) for 1 hour at RT on a rocking table. The membranes were incubated overnight at 4°C with anti-VEGFR2 (1:1,000, Cell Signaling Technology, cat. #9698, RRID: AB_11178792), anti-VEGFR2 pY1175 (1:500, Cell Signaling Technology, cat. #3770, RRID: AB_1642326), anti-ERK1/2 (1:2,000, Cell Signaling Technology, cat. #4695, RRID: AB_390779), anti-ERK1/2 pT202/pY204 (1:2,000, Cell Signaling Technology, cat. #4370, RRID: AB_2315112), anti-AKT (1:2,000, Cell Signaling Technology, cat. #9272, RRID: AB_329827), anti-AKT pS473 (1:2,000, Cell Signaling Technology, cat. #9271, RRID: AB_329825), and anti–β-actin (1:2,000, Cell Signaling Technology, cat. #4967, RRID: AB_330288). All primary antibodies were diluted in 1× TBST with 5% bovine serum albumin (Bio-Rad). Membranes were washed with 1× TBST three times for 5 minutes at RT and later incubated with secondary anti-rabbit IgG HRP-linked antibody (1:1,000 or 1:2,000, Cell Signaling Technology cat. #7074, RRID: AB_2099233) dissolved in 5% skim milk for 1 hour at RT. Membranes were washed with 1× TBST three times for 10 minutes at RT. Protein was visualized by Clarity ECL (Bio-Rad) and medical-grade X-ray film (Carestream).

### ODX generation

For the generation of an ODX model, 3 to 4 domes, totaling approximately 1 × 10^6^ cells, were combined and implanted in the subcutaneous pocket at the flank of nonobese diabetic severe combined immune-deficient (NOD SCID) female mice (23). Tumor growth was measured twice weekly with a caliper for the length (L, largest diameter) and its perpendicular width (W), including skin fold. The volume was calculated using the formula V = W^2^ × L/2. Tumors were harvested once they reached a humane endpoint size of 1.5 cm in largest diameter, divided for further studies, and cryobanked at the Princess Margaret Living Biobank PDX (PMLBPDX) Core (RRID: SCR_027523).

Animal care followed the guidelines of UHN policies and the guidelines of the Canadian Council on Animal Care and was consistent with ARRIVE guidelines for study design (24).

### *In vivo* drug screening

*In vivo* drug efficacy was evaluated on the OPTO.85 ODX mouse replicates. These were generated from donor tumor fragments derived from implanting three organoid domes at the subcutaneous flank of NOD SCID female mice and grown to a size of 1 cm largest diameter. Research grade drugs were purchased from UHN Shanghai Research & Development Co., Ltd; BKM120 (CAS#944396070, Lot#20200212) and cediranib (CAS#288383200, Lot#20240223). When tumor replicates averaged 100 mm^3^, mice were randomized (*n* = 5/group) into total four groups for daily oral gavage with the following agents: vehicle (cediranib’s solvent: DMSO 5%, PEG300 50%, Tween-20 5%, and H2O 40%), VEGFR inhibitor cediranib (6 mg/kg), pan-class 1 PI3K inhibitor BKM120 [50 mg/kg, solvent NMP (1-methyl-2-pyrrolidone)/PEG300 at 10/90 v/v], and the combined of BKM120 and cediranib was given sequentially. Tumor sizes and mouse body weights were measured twice weekly.

## Results

### Case presentation

**Figure 1.**
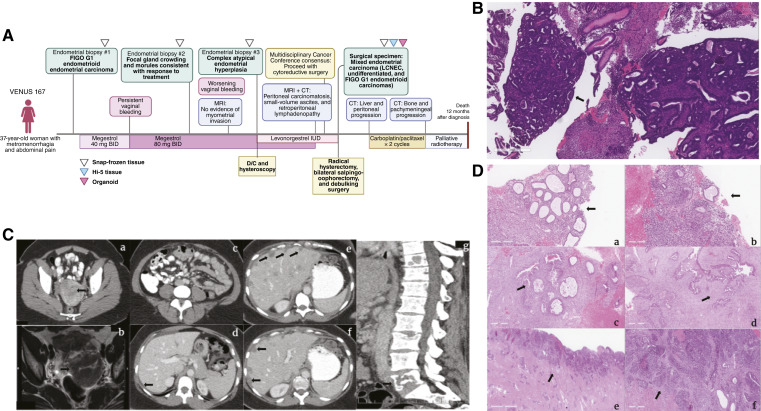
Clinical case description. **A,** Clinical case timeline. BID, twice a day. **B,** Hematoxylin and eosin (H&E) image of initial biopsy, **C,** Imaging timeline (a = initial CT scan before initiation of treatment; b = pelvis MRI; c and d = presurgical CT scans; e and f = postsurgical CT scans; g = CT spine after completion of 2 cycles of chemotherapy) **D,** H&E images of tissue samples on treatment (a and b = 3 months after biopsy; c and d = 6 months after biopsy; e = hysterectomy specimen showing extensive disease extending throughout the corpus; and f = predominantly solid and discohesive high-grade tumor with area of necrosis). Scale bar, 300 μm.

A 37-year-old woman (VENUS 167) presented with metrorrhagia and abdominal pain (Fig. 1A). Written informed consent was obtained from the patient for participation in the study and use of clinical samples. At initial diagnosis, endometrial biopsy showed FIGO grade 1 endometrioid endometrial adenocarcinoma (Fig. 1B). She was started on megestrol 40 mg twice a day as a fertility-sparing treatment. Pathology review was consistent with FIGO grade 1 endometrial cancer, MMR-proficient (Supplementary Fig. S1). Her megestrol dose was increased to 80 mg twice a day, and MRI and CT scans showed no evidence of distant metastasis or myometrial invasion [Fig. 1C (a and b)]. Three-month follow-up biopsy showed a good treatment response with focal residual gland crowding and morules [Fig. 1D (a and b)].

Despite continuing megestrol for another 3 months, her bleeding worsened. A repeat biopsy revealed persistent atypical endometrial hyperplasia [Fig. 1D (c and d)]. Given persistent vaginal bleeding, restaging imaging was performed, which revealed peritoneal carcinomatosis, new small-volume ascites, and retroperitoneal lymphadenopathy [Fig. 1C (c and d)]. Her case was discussed at the Multidisciplinary Cancer Conference, and surgical treatment was recommended. She subsequently underwent R1 cytoreductive surgery, including modified radical hysterectomy, bilateral salpingo-oophorectomy, liver parenchymal resection, omentectomy, appendectomy, and pelvic lymphadenectomy.

Pathologic examination revealed carcinoma admixed with neuroendocrine composed of large cell neuroendocrine carcinoma (LCNEC, 70%), undifferentiated carcinoma (20%–30%), and FIGO grade 1 endometrioid carcinoma (<1%). Hysterectomy specimen showed extensive involvement of the corpus [Fig. 1D (e)], with predominantly solid, discohesive high-grade tumor and areas of necrosis [Fig. 1D (f)]. IHC showed intact MMR status; negativity for ER and PR receptors; wild-type (WT) p53; E-cadherin positivity in the LCNEC component with loss in the undifferentiated component; patchy synaptophysin positivity mainly in LCNEC; and diffuse AE1/3 keratin positivity (Supplementary Fig. S2A) LCNEC was the predominant pattern, followed by undifferentiated carcinoma.

Her disease progressed 1 month postoperatively, with new liver and peritoneal metastases [Fig. 1C (e and f)]. She received two cycles of palliative carboplatin and paclitaxel but was hospitalized with worsening pain. Imaging revealed further progression with lytic bone lesions, especially in the lumbar spine [Fig. 1C (g)]. Radiation oncology was consulted, but before palliative treatment could begin, she presented with new neurologic symptoms. Brain MRI showed multiple bone metastases involving the skull base, calvarium, and clivus, with diffuse multifocal pachymeningeal enhancement. She underwent whole-brain radiotherapy and lumbar spine radiation. Despite the treatment, her condition deteriorated, and she passed away.

Next-generation sequencing (Hi5) on surgical tumor tissue identified oncogenic alterations in *PIK3CA* (p.H1047R), *ARID1A* (p.Q1452Rfs*29), and *CTNNB1* (p.S37Y), with a *TP53* WT status (Supplementary Table S1).

### Organoid and xenograft modeling

**Figure 2.**
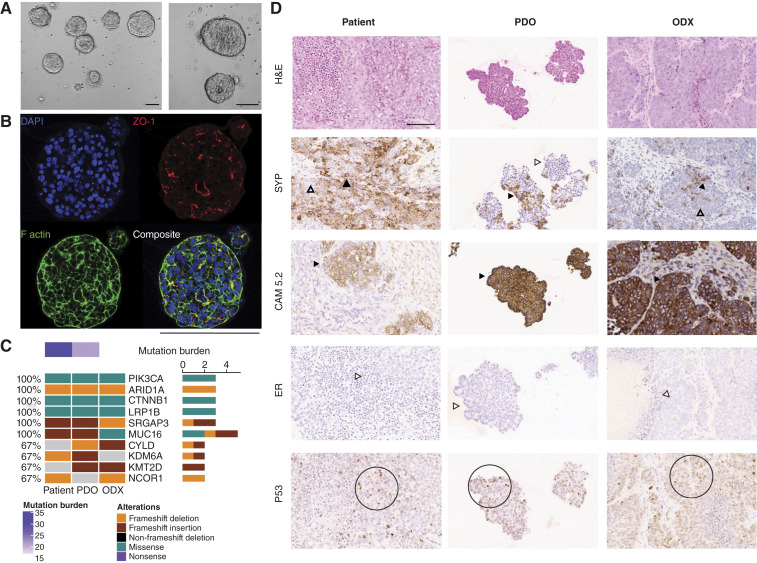
VENUS 167–derived organoid culture, OPTO.85, expands long-term *in vitro* and *in vivo* while maintaining the histologic architecture of the patient tumor. **A,** Brightfield images of OPTO.85 organoid culture. **B,** Immunofluorescent staining of the organoid model. Nuclei are stained with 4′,6-diamidino-2-phenylindole (DAPI; blue). Tight junctions are labeled with zonula occludens-1 (ZO-1; red). Actin filaments are visualized with F-actin (filamentous actin; green). **C,** Oncoprint displaying the concordance of genomic alterations in the most mutated endometrial carcinoma genes across patient tumor, PDO, and ODX tissues. **D,** Hematoxylin and eosin (H&E) and IHC staining of tumor sections, ODX, and PDO exhibit consistent morphologies and biomarker expression. Biomarkers include synaptophysin (SYP), cytokeratin (CAM5.2), neural cell adhesion molecule 1 (NCAM1), and tumor protein 53 (P53). Scale bar, 100 μm. White arrowheads indicate negative staining, and black arrowheads indicate positive staining.

A PDO model (OPTO.85) and a corresponding ODX model were generated from the VENUS 167 surgical specimen at progression on hormonal therapy (Fig. 2). The OPTO.85 organoid formed solid clusters of cells with compact, irregular organization and a thick epithelial layer (Fig. 2A). Immunofluorescence staining with zonula occludens-1 showed localization on the surface facing the spheroid-enclosed lumen (apical-in; Fig. 2B). The organoid exhibited rapid expansion and sustained long-term growth, requiring weekly passaging at a 1:6 splitting ratio, indicating robust proliferation and cellular stability (Supplementary Fig. S3A). Implantation of OPTO.85 in NOD SCID mice resulted in rapid tumor growth, with an average latency of 0.7 months (Supplementary Fig. S3B).

Histopathologic analysis confirmed that both the OPTO.85 PDO and ODX models retained key morphologic characteristics of the original tumor (Fig. 2D). The histology of the organoids showed clusters of epithelioid cells with a nested and pseudopapillary architecture, moderate nuclear pleomorphism, and focal cytoplasmic vacuolization. The PDX histology demonstrated similar solid epithelioid nests with central necrosis, moderate nuclear pleomorphism with vesicular chromatin, and prominent nucleoli. No gland formation or squamous differentiation was observed.

IHC performed on both organoid and xenograft samples showed positivity for CAM5.2 and patchy synaptophysin, with WT p53 expression (Fig. 2D). ER was negative (Fig. 2D), and both PR and PAX8 were negative (Supplementary Fig. S2B). The overall morphologic and IHC profiles of the organoid and xenograft closely matched those of the parent tumor (Fig. 2D).

### Genomic analysis and drug testing reveals PI3K driver mutation

To assess genomic concordance between the OPTO.85 PDO and ODX models and the VENUS 167 tumor, we performed WES and shallow WGS on all three DNA samples. On average, more than 60% of variants and copy-number alterations identified in the VENUS 167 patient tissue were retained in the corresponding organoid and xenograft models (Supplementary Fig. S3D). WES further revealed shared mutations in PDO and ODX models, consistent with previously reported mutations found in patients with endometrial carcinoma (25). These included alterations in *PIK3CA*, *ARID1A*, *CTNNB1*, *LRP1B*, *SRGAP3*, and *MUC16* (Fig. 2C; Supplementary Table S1).

The OPTO.85 PDO model showed resistance to megestrol, mirroring the patient’s clinical resistance (Supplementary Fig. S3C), suggesting that PDOs can inform drug-sensitivity testing. As *PIK3CA* mutations are known therapeutic targets and were identified in both the patient tumor and derived models, we hypothesized that the OPTO.85 PDO would be sensitive to PI3K inhibitors. To this end, we compared the drug sensitivities of OPTO.85 and SKOV-3, both carrying the *PIK3CA*^H1047R^ mutation, across multiple PI3K inhibitors (Supplementary Fig. S4). Both models exhibited similar sensitivity to all PI3K inhibitors tested.

### ATAC- and RNA-seq identify potential vulnerabilities to VEGF inhibition

To assess the genomic and transcriptomic landscape of OPTO.85 organoids, we performed RNA-seq and ATAC-seq and compared OPTO.85 with a panel of high-grade serous ovarian cancer organoids. We identified 17,037 ATAC-seq peaks that were shared between all OPTO.85 and HGSOC samples (Supplementary Fig. S5A; *Shared*) and 28,856 peaks specific to OPTO.85 samples (Supplementary Fig. S5A; *OPTO.85*). Annotation of the shared and OPTO.85-specific peaks showed that most shared peaks were annotated to promoter regions (within 1 kb of the transcription start site) whereas the majority of OPTO.85-specific peaks were annotated to intronic features (Supplementary Fig. S5A). Interestingly, the top enriched pathways identified using the GREAT analysis revealed enrichment for pathways related to the regulation of the VEGF pathway (Supplementary Fig. S5B) suggesting that genomic regions associated with these pathways are more accessible in OPTO.85.

Differential gene expression analysis of RNA-seq data revealed 784 differentially expressed genes (FDR <0.05 and log_2_ FC >1) upregulated in OPTO.85 compared with HGSOC (Supplementary Fig. S5C). GO enrichment analysis revealed activation of developmental pathways, including the Wnt signaling pathway (Supplementary Figs. S5D and S6). Additionally, single-sample gene set enrichment analysis of genes linked to wound healing and VEGF pathways showed enrichment in OPTO.85 gene expression (Supplementary Fig. S7). Together, these findings suggest that VEGF pathway activation is a defining feature of OPTO.85 and may represent a specific therapeutic target.

### PDO high-throughput drug screening reveals vulnerability to VEGF inhibition

**Figure 3.**
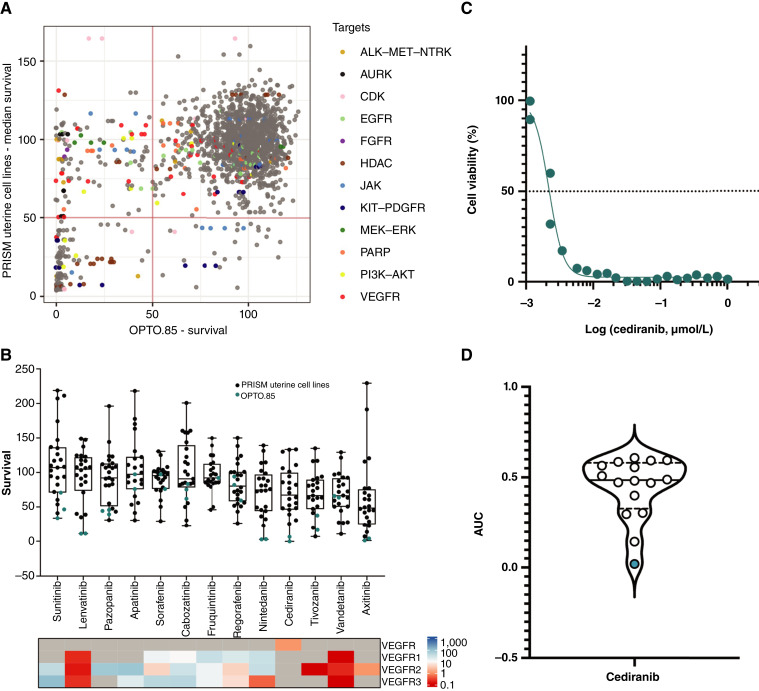
High-throughput screening of OPTO.85 PDO identifies sensitivity to the VEGFR inhibitor cediranib. **A,** Scatter plot comparing the drug response of OPTO.85 with the median response in PRISM’s uterus–endometrial cell lines. **B,** Boxplot showing increased sensitivity of the OPTO.85 PDO model to kinase inhibitors with higher selectivity for VEGFR. **C,** OPTO.85 PDO model shows sensitivity to the VEGFR inhibitor cediranib. **D,** AUC plot depicting cediranib drug–response differences between OPTO.85 PDO and HGSOC PDO models. The green dot represents OPTO.85 PDO.

High-throughput organoid drug screening identified sensitivities unique to the carcinoma’s aggressive transformation. A total of 3,372 compounds—including FDA-approved drugs, kinase inhibitors, epigenetic modulators, and tool compounds—were screened at 2.5 μmol/L to identify pathway vulnerabilities. Hits were defined as those causing >50% cell killing. The overall hit rate was 21% (723/3,372), including common cytotoxic chemotherapies, HDAC inhibitors, proteasome inhibitors, and others typically seen in such screens (Supplementary Fig. S8; Supplementary Table S2). To identify selective agents for OPTO.85, we focused on 2,837 compounds overlapping with the well-annotated PRISM drug screen dataset (14). Comparing the drug response landscape to the median response in PRISM’s uterus–endometrial cell lines (PRISM-UE, *n* = 23) identified 241 selective hits (Fig. 3A). Although not effective in the median PRISM-UE model, all 241 hits showed activity in at least one line, indicating efficacy in a subset of endometrial carcinoma. To focus on drugs with clinical viability, we excluded drugs with the following characteristics from further testing: (i) drugs in the preclinical stage of development (*n* = 54); (ii) drugs known to be unacceptably toxic at plasma concentrations in the μmol/L range, such as statins and ion channel inhibitors (*n* = 29); (iii) common cytotoxic chemotherapies which have been extensively tested in cancer (*n* = 23); and (iv) cell-cycle agents such as Aurora kinase inhibitors which have performed poorly in clinical trials (*n* = 23). The remaining hits were enriched for VEGFR inhibitors (*n* = 15; 8 unique) and PI3K–AKT pathway inhibitors (*n* = 12; 10 unique). Notably, sensitivity of OPTO.85 to VEGFR inhibitors was significantly correlated with drug specificity (IC_50_) for VEGFR2 (Fig. 3B; Supplementary Fig. S9). High sensitivity to cediranib, which showed the highest killing in the OPTO.85 model and was relatively ineffective in PRISM-UE lines, was confirmed via dose–response curve and AUC analysis (Fig. 3C and D).

### BKM120 and cediranib demonstrate *in vitro* and *in vivo* synergistic effects

**Figure 4.**
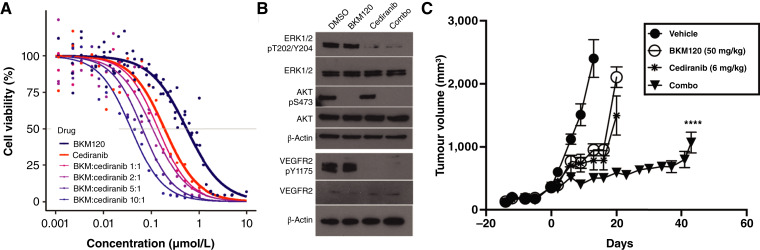
BKM120 and cediranib synergistically inhibit *in vitro* and *in vivo* growth of VENUS167 models. **A,** The combination of BKM120 and cediranib at varying concentrations demonstrates a synergistic effect in OPTO.85 organoids. Drug–response curves are based on 21-point concentration data. **B,** Targeted inhibition of VEGFR and AKT signaling pathways using single agents (0.1 μmol/L cediranib and 1 μmol/L BKM120) and combination treatment for 4 hours. **C,***In vivo* confirmation of the effects of BKM120 (50 mg/kg) and cediranib (6 mg/kg) in OPTO.85 ODX models. *N* = 5 mice per group. Error bars represent the standard error of the mean (SEM). ****, *P* < 0.05, comparing single-agent treatment with combination therapy using a two-way analysis of variance multiple comparison test.

Given the efficacy of VEGFR inhibition in some neuroendocrine carcinomas, we decided to further investigate this pathway. Based on our findings that OPTO.85 may be vulnerable to both PI3K and VEGFR inhibitors, we investigated whether these inhibitors act synergistically. To test this, we treated the OPTO.85 organoid model with dose-gradient combinations of BKM120 (PI3Ki) and cediranib (VEGFRi; Fig. 4A). Our results showed a leftward shift in the drug sensitivity curve across all tested combinations *in vitro*, showing additive and synergistic effects by Bliss and highest single agent metrics (Supplementary Fig. S10).

Western blot analysis confirmed that BKM120 strongly inhibited pAKT-S473, whereas cediranib significantly reduced pERK1/2-T202/Y204 and pVEGFR2-Y1175 levels. The combination of BKM120 and cediranib further suppressed all these phosphorylated proteins in OPTO.85 organoids (Fig. 4B).

To assess whether cediranib and BKM120 synergistically suppress tumor growth *in vivo*, we treated the OPTO.85 ODX model with BKM120, cediranib, or their combination (Fig. 4C). Both single-agent treatments reduced tumor size, whereas the combination treatment led to greater tumor inhibition compared with either agent alone (Fig. 4C).

## Discussion

Accurate grading, histologic typing, and molecular characterization of endometrial cancer are crucial, as molecular-driven treatments have become a cornerstone of personalized medicine. However, challenging diagnostic patterns necessitate further investigation and collaboration among a multidisciplinary team to ensure precise diagnosis (3). This case report describes an unusual and highly aggressive chemoresistant mixed endometrial carcinoma with three distinct histologic patterns (neuroendocrine, undifferentiated, and G1 endometrioid carcinomas).

Mixed endometrial carcinomas are high-grade neoplasms with a poor prognosis, and limited data are available as they are a unique and relatively understudied subtype of endometrial cancer (25, 26). The predominant LCNEC component is highly unusual with approximately less than 40 cases reported to date as a single histologic pattern of endometrial cancer (26). A recent review identified 20 cases of mixed LCNEC of the endometrium; however, none was associated with undifferentiated carcinoma (27), and the presence of this predominant neuroendocrine component was prognostically significant (3). In our case, LCNEC coexisted with two other morphologies representing a mixed carcinoma which was probably related to the aggressive behavior of the cancer progression after hormone treatment.

Mixed endometrial carcinomas management is particularly challenging due to the high variability of subtypes, thus requiring an individualized approach (28). In this case, the patient had a poor response to hormone and chemotherapeutic agents, leading to a fatal outcome within a short period.

Histopathologic and genomic analyses confirmed preservation of key morphologic and molecular features in OPTO.85 patient-derived models. Molecular profiling revealed enrichment in the VEGF pathway. *VEGFR*, which plays a central role in angiogenesis, is highly expressed in endometrial LCNEC, identifying it as a potential therapeutic target (29, 30). Modest efficacy (10%–15%) has been reported across different single-agent antiangiogenic agents (31). Cediranib, a VEGF receptor tyrosine kinase inhibitor, demonstrated a 12.5% response rate in patients with recurrent endometrial cancer (*n* = 48; ref. 32). Also, cediranib inhibited tumor growth in OPTO.85 models, supporting its potential antitumor activity in mixed endometrial carcinoma.

*PIK3CA* was also identified as a targetable oncogenic alteration, and the high-throughput drug screening showed this as a potential driver mutation given the sensibility to diverse PI3K inhibitors. Novel inhibitors of this pathway, such as pilaralisib, alpelisib, and buparlisib, have been studied previously as monotherapy, and other inhibitors have been FDA-approved for other solid and hematologic malignancies (33–35). The GINECO group reported that the rate of nonprogression at 2 months was 70% for those with advanced or recurrent high-grade endometrial cancer treated with BKM120 and 60% at 3 months in the low-grade group (*n* = 24; ref. 34). However, treatment with single-agent PI3K inhibitors has been limited primarily due to drug resistance and toxicity (34, 36). Overcoming drug-induced toxicity is a current challenge of utilizing tyrosine kinase inhibitors that will likely require development of newer generations of drugs to minimize off-target effects and increase targeted availability, understanding the mechanisms to better mitigate adverse effects and dose reduction while ensuring on-target biomarker efficacy (37).

The loss of the tumor-suppressor *ARID1A* may underlie aggressive behavior and treatment resistance in endometrial carcinomas, particularly through epigenetic remodeling and angiogenic activation. In this case, both the patient’s tumor and derived models harbored a rare *ARID1A* frameshift deletion (p.Q1452Rfs*29), predicted to cause functional loss. Although its role in cancer is not fully defined, ARID1A deficiency in hepatocellular carcinoma has been shown to induce ectopic expression of angiopoietin-2 (ANG2) by increasing H3K27ac modification at the *ANG2* gene locus, promoting aberrant angiogenesis and sensitizing tumors to VEGFR inhibition (38). These findings align with the VEGF pathway enrichment observed in this case and support the rationale for antiangiogenic therapy, which showed efficacy in patient-derived models.

Beyond the possible epigenetic activation of angiogenic programs associated with ARID1A loss, VEGF and PI3K signaling are also interconnected at multiple nodes that help explain the synergistic response observed with combined VEGFR and PI3K inhibition. In endometrial carcinoma, the PI3K/AKT/mTOR pathway is frequently activated and functions downstream of multiple receptor tyrosine kinases, including those involved in growth factor and angiogenic signaling, providing a biological basis for convergence between VEGF and PI3K pathway activation (39). Although not directly assessed in this study, PI3K/AKT activation has been shown to enhance HIF1a stability and increase VEGFR transcription in endometrial and other cancers (40, 41), further linking these pathways. Together, these interactions offer a possible mechanistic explanation for the enhanced response observed with combined VEGFR and PI3K inhibition in OPTO.85.

An important limitation of this study is that OPTO.85 RNA-seq and ATAC-seq data were compared with HGSOC organoid models rather than to endometrial cancer organoids. At present, no publicly available endometrial cancer organoid datasets include matched bulk RNA-seq, ATAC-seq, and HST profiles generated under comparable culturing conditions. Given this constraint, OPTO.85 and the HGSOC organoid models were derived and processed using the same media formulations, sequencing, and drug screening pipelines, minimizing technical batch effects. Additionally, although anatomically distinct, HGSOC and p53-abnormal endometrial carcinoma share VEGF-driven angiogenic signaling and show similar patterns to bevacizumab sensitivity (42, 43). Interestingly, OPTO.85 is p53 WT yet highly VEGF-dependent, indicating that angiogenic activation in this model may arise through mechanisms distinct from those described for p53-abnormal HGSOC and endometrial cancer tumors. We also functionally validated VEGF enrichment through high-throughput drug screening and integration with publicly available endometrial cancer cell lines drug response datasets.

Both VEGF and PI3K pathways are promising targets in endometrial cancer; however, the development of effective targeted therapies has been limited by safety concerns and treatment resistance. Continued innovation in drug design, patient selection, and alternative combinations are an active area of research (44). These findings highlight the value of comprehensive molecular profiling and patient-derived models identifying actionable pathways and supporting preclinical testing of molecularly informed therapies in rare, recurrent gynecologic malignancies with poor response to traditional treatments.

### Conclusions

Mixed endometrial cancer is an uncommon gynecologic malignancy with an aggressive course that often poses a challenge to diagnose and treat. The rare combination patterns (LCNEC, undifferentiated carcinoma, and G1 endometrioid carcinoma) of this case exposes the complexity of therapeutic decisions and the necessity of additional tools to identify its genomic landscape and personalize treatment. By integrating a comprehensive molecular analysis and drug screen in corresponding patient-derived models, we demonstrated that *PIK3CA* mutation was an oncogenic driver of tumor growth and the VEGF pathway was enriched. The combined inhibition of these pathways synergistically decreased growth compared with single agents. Our preclinical results support the rationale that improved cancer treatment efficacy could be achieved if a combination of therapeutic agents could be used to inhibit the multiple altered molecular pathways contributing to tumor growth in these rare forms of endometrial cancer.

## Supplementary Material

Supplementary Table 1 VENUS 167 Supplementary Table 1 - WES and CNV Data

Supplementary Table 2 VENUS 167 Supplementary Table 2-HTS Drug Data

Supplementary Figure S1 Supplementary Figure S1.

Supplementary Figure S2 Supplementary Figure S2

Supplementary Figure S3 Supplementary Figure S3

Supplementary Figure S4 Supplementary Figure S4

Supplementary Figure S5 Supplementary Figure S5

Supplementary Figure S6 Supplementary Figure S6

Supplementary Figure S7 Supplementary Figure S7

Supplementary Figure S8 Supplementary Figure S8

Supplementary Figure S9 Supplementary Figure S9

Supplementary Figure S10 Supplementary Figure S10

## Data Availability

The next-generation sequencing data generated in this study have been deposited to European Genome-phenome Archive (https://ega-archive.org/) under study ID: EGAS50000001665. The data are available under restricted access due to laws to protect the privacy of patients in alignment with UHN REB approvals and individual patient informed consent forms. Access to the data can be obtained by qualified researchers as part of an academic or industry collaboration. Requests should include a research proposal indicating the intended use of data and planned analyses. Requests will be reviewed typically within 2 weeks by the UHN Data Access Committee (DAC) and should be made by using the DAC ID: EGAC00001000912.

## References

[1] Berek JS , Matias-Guiu X, Creutzberg C, Fotopoulou C, Gaffney D, Kehoe S, . FIGO staging of endometrial cancer: 2023. Int J Gynecol Obstet 2023;162:383–94. 10.1002/ijgo.14923 37337978

[2] Concin N , Matias-Guiu X, Vergote I, Cibula D, Mirza MR, Marnitz S, . ESGO/ESTRO/ESP guidelines for the management of patients with endometrial carcinoma. Int J Gynecol Cancer 2021;31:12–39. 33397713 10.1136/ijgc-2020-002230

[3] Saglam O . Uncommon morphologic types of endometrial cancer and their mimickers: how much does molecular classification improve the practice for challenging cases? Life (Basel) 2024;14:387. 38541711 10.3390/life14030387 PMC10971728

[4] Arciuolo D , Travaglino A, Raffone A, Raimondo D, Santoro A, Russo D, . TCGA molecular prognostic groups of endometrial carcinoma: current knowledge and future perspectives. Int J Mol Sci 2022;23:11684. 36232987 10.3390/ijms231911684 PMC9569906

[5] Hamilton SN , Tinker AV, Kwon J, Lim P, Kong I, Sihra S, . Treatment and outcomes in undifferentiated and dedifferentiated endometrial carcinoma. J Gynecol Oncol 2022;33:e25. 35128856 10.3802/jgo.2022.33.e25 PMC9024191

[6] Spence T , Stickle N, Yu C, Chow H, Feilotter H, Lo B, . Inter-laboratory proficiency testing scheme for tumour next-generation sequencing in Ontario: a pilot study. Curr Oncol 2019;26:e717–32. 31896942 10.3747/co.26.5379 PMC6927773

[7] Schlechtweg K , Chen L, St Clair CM, Tergas AI, Khoury-Collado F, Hou JY, . Neuroendocrine carcinoma of the endometrium: disease course, treatment, and outcomes. Gynecol Oncol 2019;155:254–61. 31519319 10.1016/j.ygyno.2019.09.004

[8] Li H , Durbin R. Fast and accurate short read alignment with Burrows–Wheeler transform. Bioinformatics 2009;25:1754–60. 19451168 10.1093/bioinformatics/btp324 PMC2705234

[9] McKenna A , Hanna M, Banks E, Sivachenko A, Cibulskis K, Kernytsky A, . The Genome Analysis Toolkit: a MapReduce framework for analyzing next-generation DNA sequencing data. Genome Res 2010;20:1297–303. 20644199 10.1101/gr.107524.110 PMC2928508

[10] Koboldt DC , Zhang Q, Larson DE, Shen D, McLellan MD, Lin L, . VarScan 2: somatic mutation and copy number alteration discovery in cancer by exome sequencing. Genome Res 2012;22:568–76. 22300766 10.1101/gr.129684.111 PMC3290792

[11] McLaren W , Gil L, Hunt SE, Riat HS, Ritchie GRS, Thormann A, . The Ensembl Variant Effect Predictor. Genome Biol 2016;17:122. 27268795 10.1186/s13059-016-0974-4 PMC4893825

[12] Boeva V , Popova T, Bleakley K, Chiche P, Cappo J, Schleiermacher G, . Control-FREEC: a tool for assessing copy number and allelic content using next-generation sequencing data. Bioinformatics 2012;28:423–5. 22155870 10.1093/bioinformatics/btr670 PMC3268243

[13] Ramírez F , Ryan DP, Grüning B, Bhardwaj V, Kilpert F, Richter AS, . deepTools2: a next generation web server for deep-sequencing data analysis. Nucleic Acids Res 2016;44:W160–5. 27079975 10.1093/nar/gkw257 PMC4987876

[14] Corsello SM , Nagari RT, Spangler RD, Rossen J, Kocak M, Bryan JG, . Discovering the anti-cancer potential of non-oncology drugs by systematic viability profiling. Nat Cancer 2020;1:235–48. 32613204 10.1038/s43018-019-0018-6 PMC7328899

[15] Dobin A , Davis CA, Schlesinger F, Drenkow J, Zaleski C, Jha S, . STAR: ultrafast universal RNA-seq aligner. Bioinformatics 2013;29:15–21. 23104886 10.1093/bioinformatics/bts635 PMC3530905

[16] Liao Y , Smyth GK, Shi W. FeatureCounts: an efficient general purpose program for assigning sequence reads to genomic features. Bioinformatics 2014;30:923–30. 24227677 10.1093/bioinformatics/btt656

[17] Love MI , Huber W, Anders S. Moderated estimation of fold change and dispersion for RNA-seq data with DESeq2. Genome Biol 2014;15:550. 25516281 10.1186/s13059-014-0550-8 PMC4302049

[18] Kolberg L , Raudvere U, Kuzmin I, Vilo J, Peterson H. gprofiler2 – an R package for gene list functional enrichment analysis and namespace conversion toolset g:profiler. F1000Res 2020;9:ELIXIR-709. 10.12688/f1000research.24956.1 PMC7859841 33564394

[19] Corces MR , Trevino AE, Hamilton EG, Greenside PG, Sinnott-Armstrong NA, Vesuna S, . An improved ATAC-seq protocol reduces background and enables interrogation of frozen tissues. Nat Methods 2017;14:959–62. 28846090 10.1038/nmeth.4396 PMC5623106

[20] Quinlan AR . BEDTools: the Swiss-army tool for genome feature analysis. Curr Protoc Bioinformatics 2014;47:11.12.1–34. 10.1002/0471250953.bi1112s47 PMC4213956 25199790

[21] Quinlan AR , Hall IM. BEDTools: a flexible suite of utilities for comparing genomic features. Bioinformatics 2010;26:841–2. 20110278 10.1093/bioinformatics/btq033 PMC2832824

[22] Yu G , Wang LG, He QY. ChIP seeker: an R/Bioconductor package for ChIP peak annotation, comparison and visualization. Bioinformatics 2015;31:2382–3. 25765347 10.1093/bioinformatics/btv145

[23] Ng SS , Tsao MS, Nicklee T, Hedley DW. Wortmannin inhibits PKB/Akt phosphorylation and promotes gemcitabine antitumor activity in orthotopic human pancreatic cancer xenografts in immunodeficient mice. Clin Cancer Res 2001;7:3269–75. 11595724

[24] Kilkenny C , Browne WJ, Cuthill IC, Emerson M, Altman DG. Improving bioscience research reporting: the arrive guidelines for reporting animal research. PLoS Biol 2010;8:e1000412. 20613859 10.1371/journal.pbio.1000412 PMC2893951

[25] Kandoth C , Schultz N, Cherniack AD, Akbani R, Liu Y, Shen H, ; Cancer Genome Atlas Research Network. Integrated genomic characterization of endometrial carcinoma. Nature 2013;497:67–73. 23636398 10.1038/nature12113 PMC3704730

[26] Yang F , Liang S, Liu C, Wei Y, Zhang L. Endometrial large cell neuroendocrine carcinoma: a case report and literature review. Gynecol Oncol Rep 2024;54:101429. 38939507 10.1016/j.gore.2024.101429 PMC11208911

[27] Gu Z , Eils R, Schlesner M. Complex heatmaps reveal patterns and correlations in multidimensional genomic data. Bioinformatics 2016;32:2847–9. 27207943 10.1093/bioinformatics/btw313

[28] Pappa C , Le Thanh V, Smyth SL, Zouridis A, Kashif A, Sadeghi N, . Mixed endometrial epithelial carcinoma: epidemiology, treatment and survival rates—a 10-year retrospective cohort study from a single institution. J Clin Med 2023;12:6373. 37835017 10.3390/jcm12196373 PMC10573791

[29] Andrini E , Marchese PV, De Biase D, Mosconi C, Siepe G, Panzuto F, . Large cell neuroendocrine carcinoma of the lung: current understanding and challenges. J Clin Med 2022;11:1461. 35268551 10.3390/jcm11051461 PMC8911276

[30] Salvo G , Gonzalez Martin A, Gonzales NR, Frumovitz M. Updates and management algorithm for neuroendocrine tumors of the uterine cervix. Int J Gynecol Cancer 2019;29:986–95. 31263021 10.1136/ijgc-2019-000504

[31] MacKay HJ , Freixinos VR, Fleming GF. Therapeutic targets and opportunities in endometrial cancer: update on endocrine therapy and nonimmunotherapy targeted options. Am Soc Clin Oncol Educ Book 2020;40:1–11. 10.1200/EDBK_280495 32239967

[32] Bender D , Sill MW, Lankes HA, Reyes HD, Darus CJ, Delmore JE, . A phase II evaluation of cediranib in the treatment of recurrent or persistent endometrial cancer: an NRG Oncology/Gynecologic Oncology Group study. Gynecol Oncol 2015;138:507–12. 26186911 10.1016/j.ygyno.2015.07.018 PMC4642817

[33] Passarelli A , Carbone V, Pignata S, Mazzeo R, Lorusso D, Scambia G, . Alpelisib for PIK3CA-mutated advanced gynecological cancers: first clues of clinical activity. Gynecol Oncol 2024;183:61–7. 38518529 10.1016/j.ygyno.2024.02.029

[34] Heudel P-E , Fabbro M, Roemer-Becuwe C, Kaminsky MC, Arnaud A, Joly F, . Phase II study of the PI3K inhibitor BKM120 in patients with advanced or recurrent endometrial carcinoma: a stratified type I-type II study from the GINECO group. Br J Cancer 2017;116:303–9. 28072765 10.1038/bjc.2016.430 PMC5294485

[35] He XP , Cao XD. Effects of intrahippocampal delta-receptors on inhibition of electroconvulsive shock by electro-acupuncture. Acta Pharmacol Sin 1989;10:197–201. 2558496

[36] Bostan I-S , Mihaila M, Roman V, Radu N, Neagu MT, Bostan M, . Landscape of endometrial cancer: molecular mechanisms, biomarkers, and target therapy. Cancers (Basel) 2024;16:2027. 38893147 10.3390/cancers16112027 PMC11171255

[37] Shyam Sunder S , Sharma UC, Pokharel S. Adverse effects of tyrosine kinase inhibitors in cancer therapy: pathophysiology, mechanisms and clinical management. Signal Transduct Target Ther 2023;8:262. 37414756 10.1038/s41392-023-01469-6 PMC10326056

[38] Hu C , Li W, Tian F, Jiang K, Liu X, Cen J, . Arid1a regulates response to anti-angiogenic therapy in advanced hepatocellular carcinoma. J Hepatol 2018;68:465–75. 29113912 10.1016/j.jhep.2017.10.028

[39] Slomovitz BM , Coleman RL. The PI3K/AKT/mTOR pathway as a therapeutic target in endometrial cancer. Clin Cancer Res 2012;18:5856–64. 23082003 10.1158/1078-0432.CCR-12-0662

[40] Ghalehbandi S , Yuzugulen J, Pranjol MZI, Pourgholami MH. The role of VEGF in cancer-induced angiogenesis and research progress of drugs targeting VEGF. Eur J Pharmacol 2023;949:175586. 36906141 10.1016/j.ejphar.2023.175586

[41] Seeber LMS , Zweemer RP, Verheijen RHM, van Diest PJ. Hypoxia-inducible factor-1 as a therapeutic target in endometrial cancer management. Obstet Gynecol Int 2010;2010:580971. 20169098 10.1155/2010/580971 PMC2821774

[42] Thiel KW , Devor EJ, Filiaci VL, Mutch D, Moxley K, Alvarez Secord A, . TP53 sequencing and p53 immunohistochemistry predict outcomes when bevacizumab is added to frontline chemotherapy in endometrial cancer: an NRG Oncology/Gynecologic Oncology Group Study. J Clin Oncol 2022;40:3289–300. 35658479 10.1200/JCO.21.02506 PMC9553389

[43] Bignotti E , Simeon V, Ardighieri L, Kuhn E, Marchini S, Califano D, . TP53 mutations and survival in ovarian carcinoma patients receiving first-line chemotherapy plus bevacizumab: results of the MITO16A/MaNGO OV-2 study. Int J Cancer 2025;156:1085–96. 39415516 10.1002/ijc.35203 PMC11701406

[44] Soberanis Pina P , Lheureux S. Novel molecular targets in endometrial cancer: mechanisms and perspectives for therapy. Biologics 2024;18:79–93. 38529411 10.2147/BTT.S369783 PMC10962462

