# Supplementary material for: Preclinical Combination Targeting VEGF and PI3K in a Rare, Aggressive Mixed Endometrial Carcinoma: An Applied Case Report
Source: Cancer Res Commun. 2026 Apr 15;6(4):832–41. doi: 10.1158/2767-9764.CRC-25-0634 (PMC13081119; doi:10.1158/2767-9764.CRC-25-0634)
Supplement: Supplementary Figure S2 [file crc-25-0634_supplementary_figure_s2_suppsf2.docx]

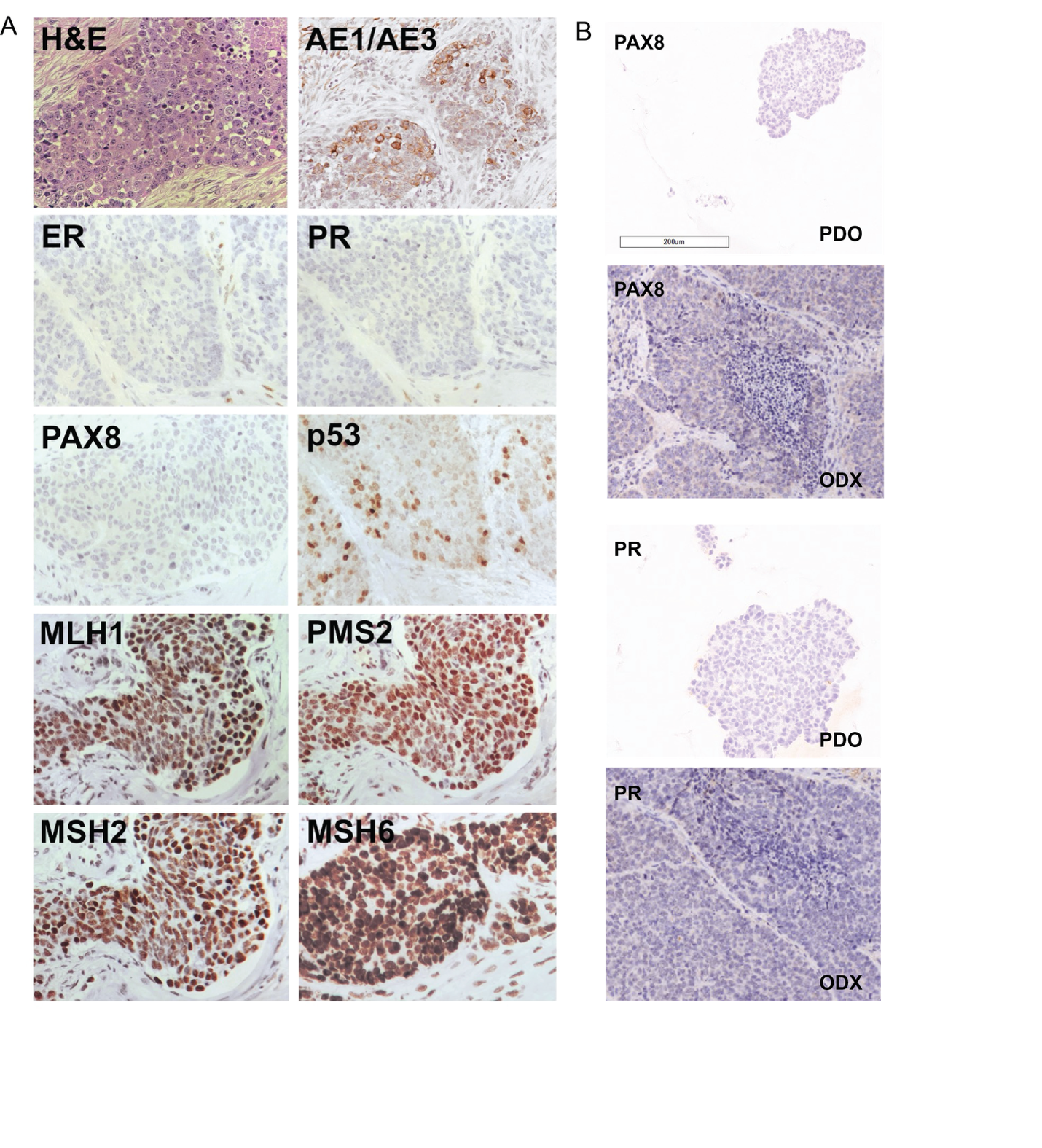
**Supplementary Figure S2:** **A.** Surgical specimen immunohistochemistry, showing patchy positivity for AE1/AE3, negative ER/PR, negative PAX8, wildtype (heterogenous) p53, and retained MLH1/PMS2/MSH2/MSH6. **B.** PDO and ODX models showing negative staining for PR and PAX0. All on-slide and internal controls stained appropriately.
