# Supplementary material for: Preclinical Combination Targeting VEGF and PI3K in a Rare, Aggressive Mixed Endometrial Carcinoma: An Applied Case Report
Source: Cancer Res Commun. 2026 Apr 15;6(4):832–41. doi: 10.1158/2767-9764.CRC-25-0634 (PMC13081119; doi:10.1158/2767-9764.CRC-25-0634)
Supplement: Supplementary Figure S3 [file crc-25-0634_supplementary_figure_s3_suppsf3.docx]

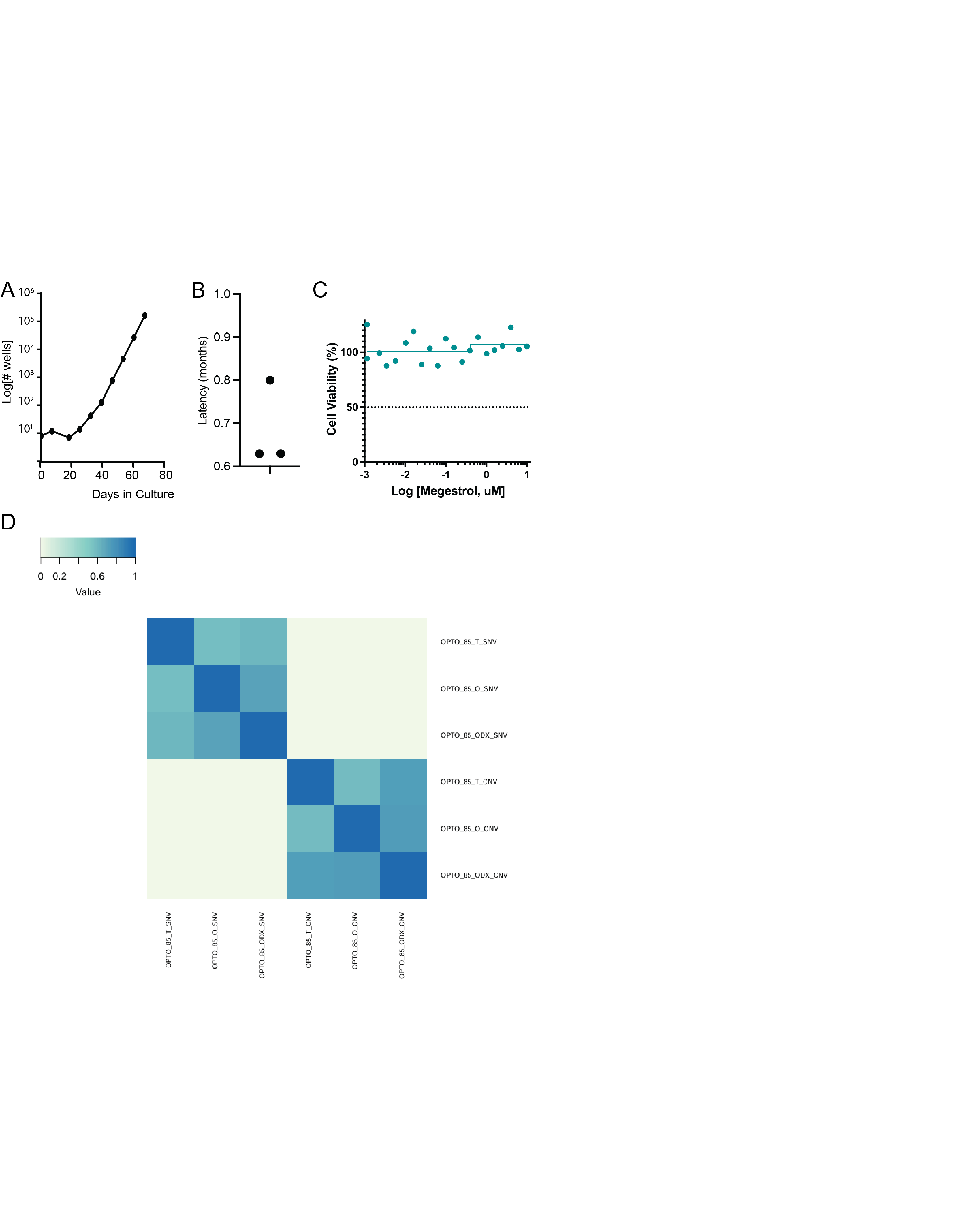


**Supplementary Figure S3:** **A.** Graph depicting exponential growth of organoids through successive passages; each data point represents a passage. Growth was calculated by plotting the time to passaging and the cumulative sum of the number of wells plated. **B.** Subcutaneous implantation of the organoid model shows tumor formation within one month. Tumor latency is defined as the time (in months) to reach a tumor volume of 1000 mm³ (n=3). **C.** OPTO.85 PDO model shows resistance to Megestrol. Data points and error bars represent the mean+SD from three independent experiments. **D.** Concordance of exonic and copy number variants between the primary tumor tissue and patient-derived models. Abbreviations: O = PDO model; T = patient tumor; ODX = organoid-derived xenograft; SNV = single nucleotide variant; CNV = copy number variant.
