# Supplementary material for: Preclinical Combination Targeting VEGF and PI3K in a Rare, Aggressive Mixed Endometrial Carcinoma: An Applied Case Report
Source: Cancer Res Commun. 2026 Apr 15;6(4):832–41. doi: 10.1158/2767-9764.CRC-25-0634 (PMC13081119; doi:10.1158/2767-9764.CRC-25-0634)
Supplement: Supplementary Figure S4 [file crc-25-0634_supplementary_figure_s4_suppsf4.docx]

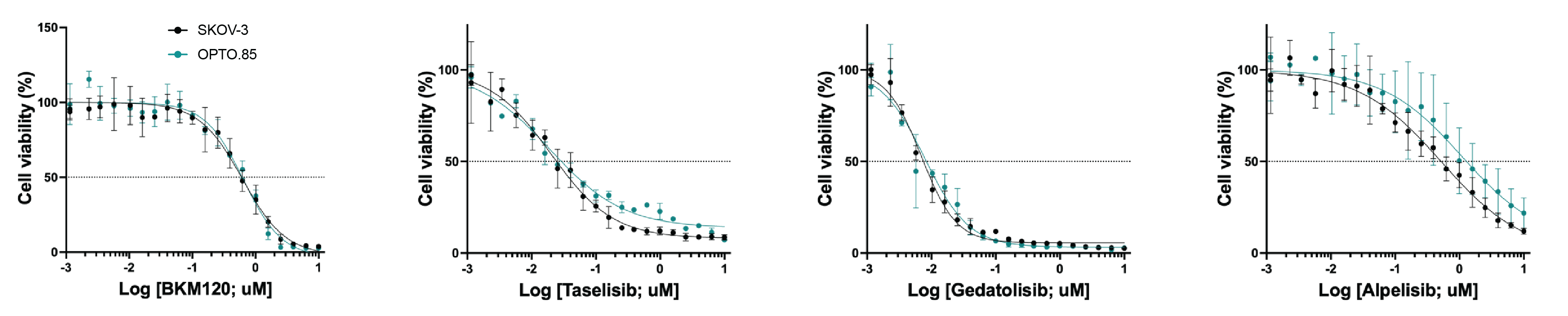


**Supplementary Figure S4: OPTO.85 PDO model and SKOV-3 cell line, both harboring *PIK3CA*^H1047R^ mutation, demonstrate sensitivity to multiple PI3K inhibitors.** Data points and error bars represent the mean+SD from three independent experiments.
