# Supplementary material for: Preclinical Combination Targeting VEGF and PI3K in a Rare, Aggressive Mixed Endometrial Carcinoma: An Applied Case Report
Source: Cancer Res Commun. 2026 Apr 15;6(4):832–41. doi: 10.1158/2767-9764.CRC-25-0634 (PMC13081119; doi:10.1158/2767-9764.CRC-25-0634)
Supplement: Supplementary Figure S5 [file crc-25-0634_supplementary_figure_s5_suppsf5.docx]

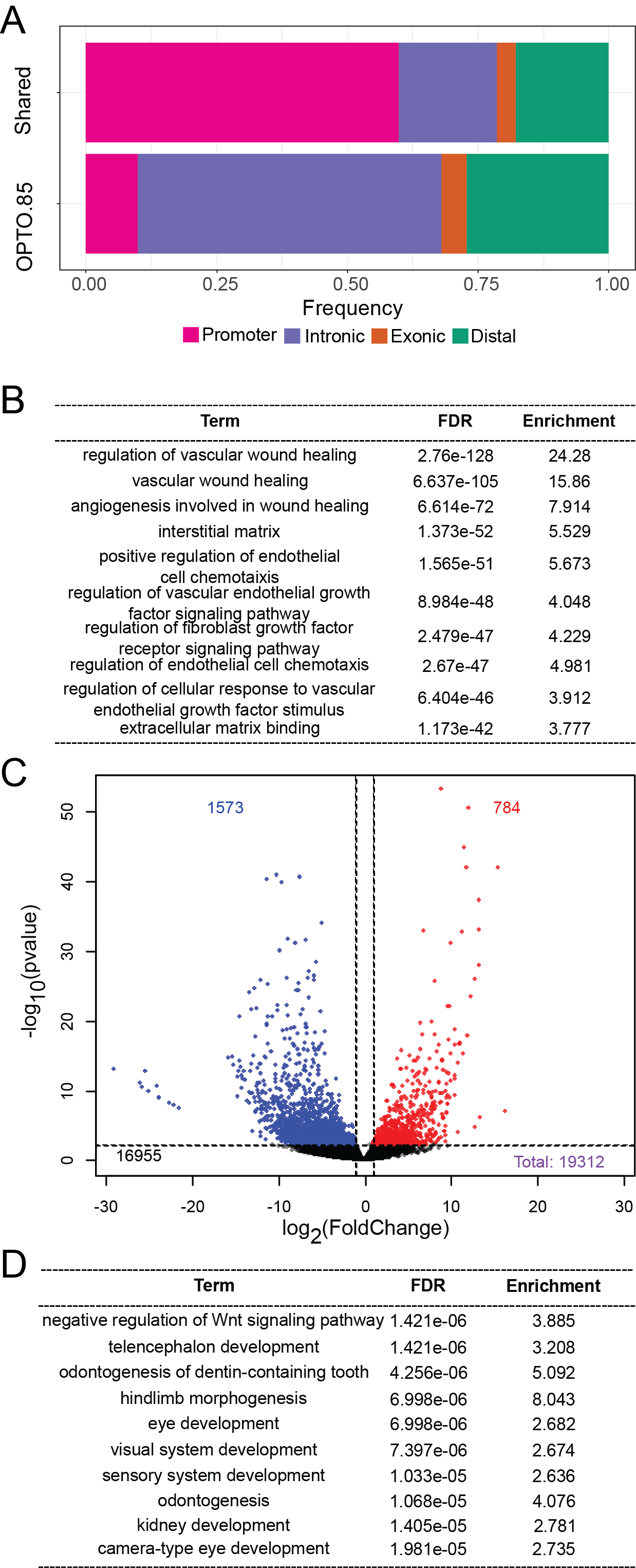


**Supplementary Figure S5: High throughput sequencing of OPTO.85 PDO compared to HGSOC PDO models reveals potential pathways for therapeutic targets. A**. Annotation of shared and OPTO.85-specific ATAC-seq peaks to genomic features: Promoter (TSS +/- 1kb), Intronic, Exonic, and Distal intergenic regions. **B.** Table of top significant GO biological processes enriched in OPTO.85-specific ATAC-seq peaks from GREAT analysis. **C.** Volcano plot showing differentially expressed genes (FDR<0.05 & |log2FoldChange| > 1) between OPTO.85 PDO and HGSOC PDO models. Red points indicate genes upregulated in OPTO.85, blue points indicate genes downregulated in OPTO.85. **D.** Table of top significant GO biological processes enriched in OPTO.85 upregulated genes.
