# Supplementary material for: Preclinical Combination Targeting VEGF and PI3K in a Rare, Aggressive Mixed Endometrial Carcinoma: An Applied Case Report
Source: Cancer Res Commun. 2026 Apr 15;6(4):832–41. doi: 10.1158/2767-9764.CRC-25-0634 (PMC13081119; doi:10.1158/2767-9764.CRC-25-0634)
Supplement: Supplementary Figure S7 [file crc-25-0634_supplementary_figure_s7_suppsf7.docx]

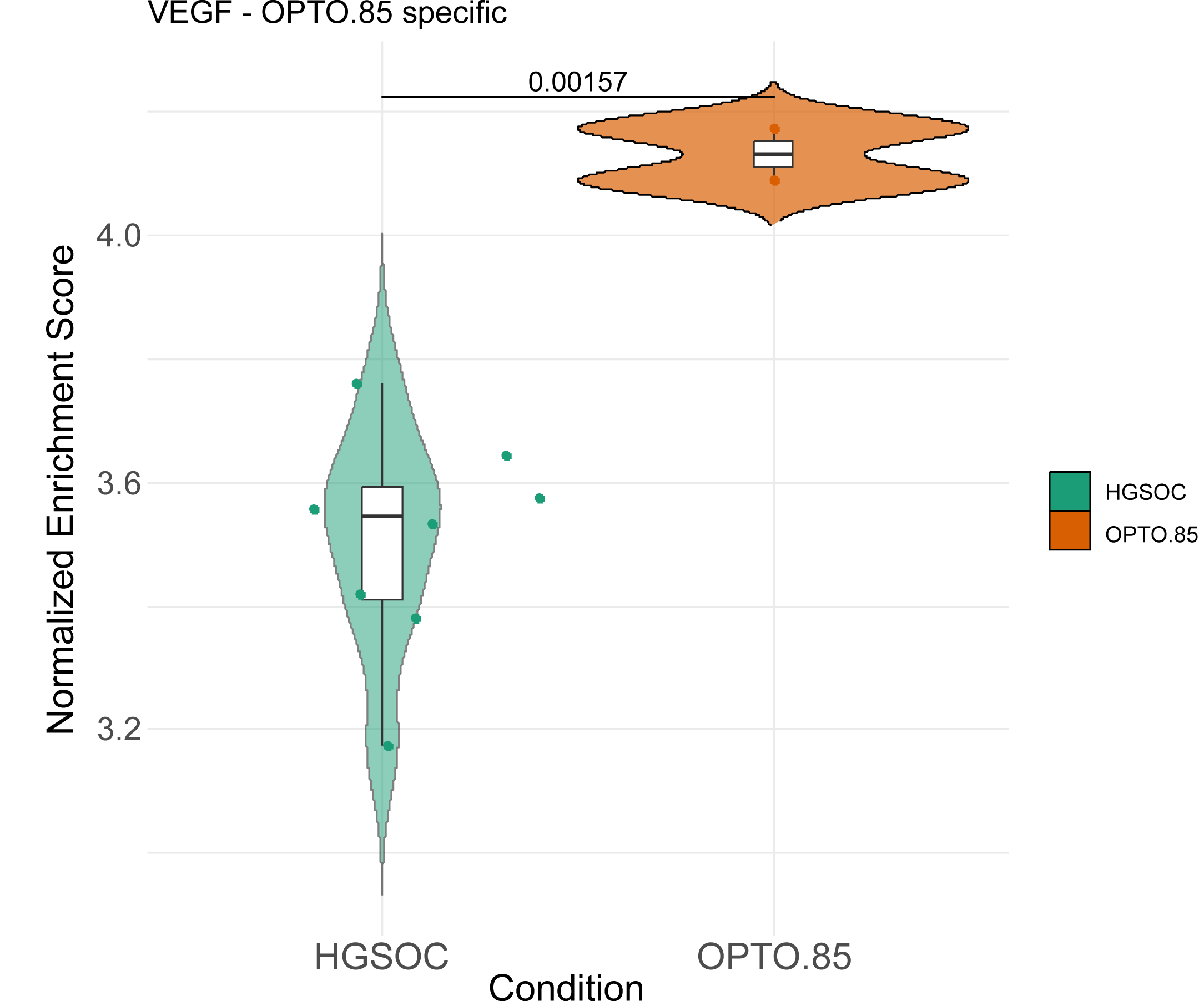


**Supplementary Figure S7.** Violin plot of single sample gene set enrichment analysis (ssGSEA) scores for expression of genes associated with VEGF-related pathways that were enriched in OPTO.85-specific ATAC-seq peaks.
