# Supplementary material for: Preclinical Combination Targeting VEGF and PI3K in a Rare, Aggressive Mixed Endometrial Carcinoma: An Applied Case Report
Source: Cancer Res Commun. 2026 Apr 15;6(4):832–41. doi: 10.1158/2767-9764.CRC-25-0634 (PMC13081119; doi:10.1158/2767-9764.CRC-25-0634)
Supplement: Supplementary Figure S8 [file crc-25-0634_supplementary_figure_s8_suppsf8.docx]

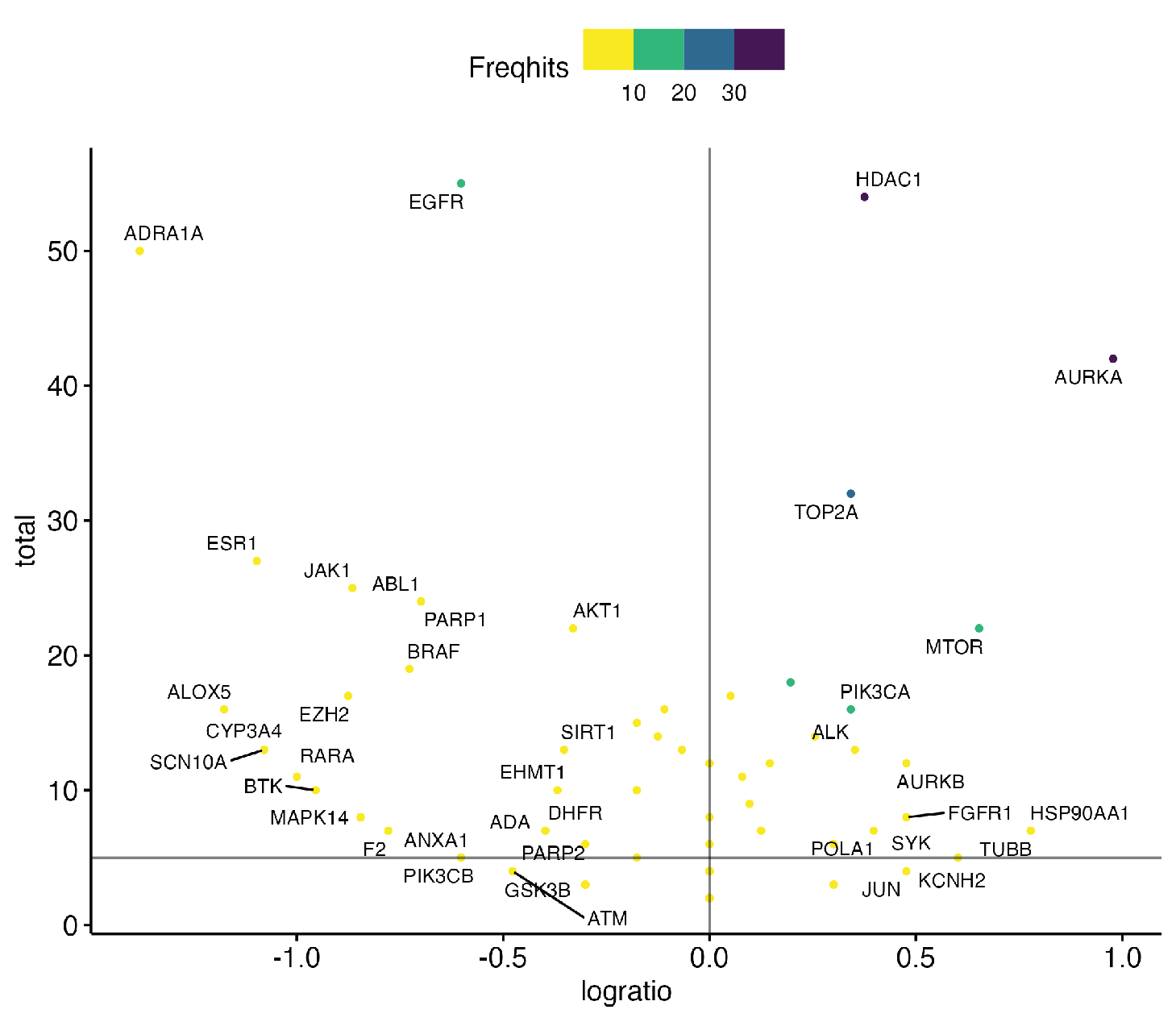


**Supplementary Figure S8.** Scatter plot showing drug hits identified in the OPTO.85 model using high-throughput drug screening. Hits are defined as drugs that reduce cell viability to less than 50%.
